# The Qingdao Preschooler Facial Expression Set: Acquisition and Validation of Chinese Children’s Facial Emotion Stimuli

**DOI:** 10.3389/fpsyg.2020.554821

**Published:** 2021-01-21

**Authors:** Jie Chen, Yulin Zhang, Guozhen Zhao

**Affiliations:** ^1^CAS Key Laboratory of Behavioral Science, Institute of Psychology, Beijing, China; ^2^Department of Psychology, University of Chinese Academy of Sciences, Beijing, China

**Keywords:** emotional stimulus, positive emotion, facial expression, preschooler, emotion perception

## Abstract

Traditional research on emotion-face processing has primarily focused on the expression of basic emotions using adult emotional face stimuli. Stimulus sets featuring child faces or emotions other than basic emotions are rare. The current study describes the acquisition and evaluation of the Qingdao Preschooler Facial Expression (QPFE) set, a facial stimulus set with images featuring 54 Chinese preschoolers’ emotion expressions. The set includes 712 standardized color photographs of six basic emotions (joy, fear, anger, sadness, surprise, and disgust), five discrete positive emotions (interest, contentment, relief, pride, and amusement), and a neutral expression. The validity of the pictures was examined based on 43 adult raters’ online evaluation, including agreement between designated emotions and raters’ labels, as well as intensity and representativeness scores. Overall, these data should contribute to the developmental and cross-cultural research on children’s emotion expressions and provide insights for future research on positive emotions.

## Introduction

Facial expression, the most prominent cue of emotion, has been studied extensively for decades within the scope of face processing, emotion perception, as well as the impact of emotion on cognition and socialization (Adolphs, 2002; Nelson et al., 2003; Todd et al., 2008). Designing a highly standardized facial stimulus set is crucial to the reliability of research on emotion expression and perception (Calvo and Lundqvist, 2008; Sabatinelli et al., 2011). Based on the six basic human emotions that were identified by Ekman et al. (1971), the first facial stimulus set of emotion expressions, pictures of facial affect (POFA), was created (Ekman and Friesen, 1976) and has been widely studied in the traditional face-emotion research. After POFA, several new stimulus sets were further developed adapting to specific research demands (Bänziger et al., 2011).

Despite the increase of stimulus sets featuring adult emotional faces, children’s facial databases remained poorly developed in the early years. However, evidence including facial discrimination ability (Macchi Cassia, 2011; Macchi Cassia et al., 2012), face responding time (Benoit et al., 2012), functional MRI (fMRI) showing brain activation (Blakemore, 2008; Hoehl et al., 2010; Marusak et al., 2013), and event-related potential (ERP) studies (Taylor et al., 2004; Leppänen et al., 2007; Miki et al., 2015) all highlighted the importance of using stimulus sets featuring children’s faces in studying peer emotion displays. Researchers started to create stimulus sets specific to children’s facial expressions in an attempt to control the age biases in face-processing (Egger et al., 2011; Dalrymple et al., 2013; LoBue and Thrasher, 2014). Every database was developed with unique features, such as children’s age and race, facial expression conditions, and expression elicitation methods (Grossard et al., 2018).

Here, we introduce a new and innovative stimulus set, the Qingdao Preschooler Facial Expression (QPFE) set, with the intention to broaden children’s facial expression database family and to deepen the emotional development research domain. QPFE contains 712 standardized frontal and profile color images of 54 Chinese children aged 5–7. It includes the six basic emotions, joy, surprise, disgust, sadness, anger, and fear, and five discrete positive emotions, amusement, pride, interest, contentment, and relief, along with a neutral expression. The innovation of our database lines in the following aspects below.

According to the own-race effect discovered by Matsumoto (1992), individuals could better understand and discriminate own- versus other-race faces. It is necessary to create facial stimulus sets specific to local cultures and ethnic groups in order to control in-group bias. Further research showed that ethnicity and culture could influence facial expression production not only in adults but also in children, especially between Asian and Western countries. Compared with European or American, Asian children were less expressive for disgust (Camras et al., 2006), sadness, and exuberance (Louie et al., 2013). To our knowledge, there is no independent Chinese children’s facial expression stimuli database to date. The widely used Chinese Facial Affective Picture System (CFAPS) developed by Gong et al. (2011) included images of few 8- to 12-year-old children posing basic expressions along with 200 adult expressers. Our database would properly fill in the gap of Chinese ethnicity, thereby adding diversity to the cross-cultural emotion-face research.

The Qingdao Preschooler Facial Expression set includes not only six basic facial expressions but also five discrete positive facial expressions, which is highly innovation. In previous studies, researchers mainly focused on the six basic emotions in facial stimulus sets, and among these, joy or happiness is the only positive emotion being featured (Egger et al., 2011; Dalrymple et al., 2013; LoBue and Thrasher, 2014). Indeed, in the early categorization of emotions, psychologists referred to positive emotions simply using the word “joy” or “happiness” (Ekman, 1992). In general, positive emotions are far less studied than negative emotions, despite that a normal person would experience positive emotions more frequently than negative emotions in a lifetime (Shiota et al., 2017). The lack of research on positive emotions is possibly due to the limited understanding of positive emotion display behaviors. For example, the Duchenne smile, which involves lifting of the lip corners and contraction of the orbicularis oculi muscles around the eyes, has previously been regarded as the one and only reliable behavioral marker of positive emotions (Mortillaro et al., 2011; Campos et al., 2013). After Fredrickson’s (1998) study on positive emotions, researchers had confirmed that positive emotions are different in many ways, such as subjective perception, cognitive appraisal, physiological arousal, as well as external expression (Griskevicius et al., 2010; Mortillaro et al., 2011; Shiota et al., 2011; Campos et al., 2013). Despite the emergence of research on positive emotions, the inclusion of newly differentiated positive emotions was still rare in facial expression stimulus sets. The very few facial stimulus sets that expand the scope of positive emotions include: UC Davis Set of Emotion Expressions (UCDSEE), developed by Tracy et al. (2009), includes “pride” as a positive emotion in addition to “happiness”; Geneva Multimodal Emotion Portrayals Core Set (GEMEP-CS), developed by Bänziger et al. (2011), contains happiness, amusement, pride, relief, admiration, and tenderness; and the Dartmouth Database of Children’s Faces, designed by Dalrymple et al. (2013), distinguishes “contentment” from “happiness” by “smile with no teeth” and “smile with teeth.”

In the QPFE, we collected facial expressions of five discrete positive emotions amusement, pride, relief, contentment, and interest. These emotions were chosen based on positive emotion classification, as well as preschooler’s comprehension ability. According to Sauter (2017), positive emotions could be preliminarily classified into four emotion families: epistemological, prosocial, savoring, and agency-approach positive emotions. Epistemological positive emotions consisting of amusement, relief, awe, and interest have been verified to contain recognizable displays *via* facial cues. Amusement was the feeling of finding things funny and was strongly linked with intense Duchenne smiles (Ambadar et al., 2009; Campos et al., 2013). Relief was the feeling when an unpleasant experience ended and usually linked with low-intensity smile, open mouth, and eye closure (Krumhuber and Scherer, 2011). Interest was the feeling of wanting to learn something more and usually linked with an open mouth (Mortillaro et al., 2011) or lip presses (Campos et al., 2013) and mild squinting or eye closure (Mortillaro et al., 2011). Pride from the agency-approach positive emotion family was the feeling when a goal that could enhance social status was completed and strongly linked with small smile, crow’s feet, parted lips, and raised chin (Tracy and Robins, 2008). Sauter (2017) named pride to be one of the strongest candidates for positive facial expressions that were specific and identifiable. Contentment from savoring positive emotion family was the feeling when one’s basic needs were satisfied and usually linked with low-intensity smiles with lips pressed (Campos et al., 2013). Other mentioned positive emotions were excluded because either they exceeded preschooler’s cognition or daily life experience, such as sexual desire, awe, and elation, or they were not reliably communicated by facial expressions, such as prosocial positive emotion family (love, compassion, gratitude, and admiration) that was reliable only in touch (Sauter, 2017). In this paper, we name amusement, pride, relief, contentment, and interest as the discrete positive emotion group, in order to distinguish them from joy or happiness in the basic emotion group. Joy was the feeling of happiness, with response to obtaining an unexpected reward, usually linked with cheek raiser and lip corner puller (Campos et al., 2013). Joy or happiness was regarded as an individual emotion in previous research that also contained other positive emotions. In GEMEP-CS (Bänziger et al., 2011), joy was presented with other positive emotions including amusement, interest, sensory pleasure, pride, relief, admiration, and tenderness. Similarly, in Mortillaro et al.’s (2011) research, joy was presented with pride, interest, and sensory pleasure.

In addition, there were research studies suggested that culture may play a major role in positive emotions signals shaping. People from individualistic cultures would value high activation positive states, whereas people from collectivistic cultures would value low activation positive states (Tsai et al., 2006). Sauter et al. (2010) discovered that basic emotions might be recognized universally, but positive emotions might communicate with culture-specific signals, and the role of social learning would vary across positive emotions. There is a necessity for cross-cultural research in the study of positive emotions, as work using other than Western samples were rare. These would increase the urgency of collecting positive facial expressions from different cultures and which in this case are Chinese children.

Among the existing children facial expression stimulus sets, two expression elicitation procedures were adopted: prototype posing and felt experience acting. Prototype posing technique was adopted by the Radboud Faces Database (Langner et al., 2010), the NIMH Child Emotional Faces Picture Set (NIMH-ChEFS) (Egger et al., 2011), and the Qingdao Preschooler Facial Expression (QPFE) set (LoBue and Thrasher, 2014). The prototype here usually referred to the facial action coding system (FACS) that was developed by Ekman and Friesen (1978). In prototype posing, expressers were instructed to coordinate specific facial action units and pose expressions based on the emotion prototypes. Usually expressers would rehearse and practice the facial muscle movements, and the whole procedure was supervised under FACS experts. No acting was required in the procedure. Despite the high reliability of prototype posing, the procedure often fails to account for the subtle individual and cultural differences in expressive styles (Bänziger et al., 2011). Felt experience acting technique was adopted by the CFAPS (Gong et al., 2011), the Developmental Emotional Faces Stimulus Set (DEFSS) (Meuwissen et al., 2017), and the Dartmouth Database of Children’s Faces (Dalrymple et al., 2013). Expressers were asked to reminisce about the specific past events associated with certain emotions and to express them vividly (Scherer and Bänziger, 2010). It was a combination of the stimulus-provoked mood induction technique and communication effect acting, required a lot of acting from expressers (Bänziger et al., 2011). The felt experience acting often results in significant expression variations and, thus, is suitable for studies with no prototypes for emotion expressions or for research on facial expressions of diverse ethnicities (Scherer and Heiner, 2007). QPFE adopted felt experience acting method considering all pros and cons mentioned above. In addition, it might be the first research to adopt felt experience acting technique in children’s positive emotion elicitation. A latest research found that children produced better facial expression on request task than on imitation task, and that positive emotion (joy) was easier to produce than negative emotions (sadness, anger) for children (Grossard et al., 2018). The current research would try to expand this to other discrete positive emotions.

The current study aimed at the acquisition and validation of a new Chinese preschoolers’ facial expression stimulus set, which includes carefully selected discrete positive emotions. The effect of using felt experience acting technique on positive emotion elicitation was considered. Moreover, the validation result would also reveal the discrimination condition of positive emotions. Considering the subtle differentiation, we suspect a lower agreement rate and higher misattribution in discrete positive expressions than in basic emotion expressions.

## Materials and Methods

### Stimulus Acquisition

#### Participants

Fifty-four preschooler (23 males, 31 females, age range 5–7 years, mean 6 years ± 10 months) participants were recruited from two art training institutes in Qingdao, Shandong Province, mainland China. Participants were all native Mandarin speakers with corrected or corrected-to-normal vision and had no history of psychiatric disorders. Information on children’s religious beliefs was not obtained due to the consideration of age. Prior to the emotion induction task, parents signed the consent form and gave permissions for further use of pictures in scientific research and analysis, as well as the survey about the basic information of the children and were informed about the safety of the emotion induction task. All participants received small gifts after the session.

#### Materials

Children’s expressions were recorded with two cameras (one Canon 6D and one Sony A72) in front of a white backdrop under the lighting of two light bulbs (1,000 W). One camera was positioned directly in front of the expresser matching eye level. The second camera was placed 45° on the right side of the expresser to capture profile facial expressions. The video recording mode was turned on throughout the procedure to capture a flow of emotion displays with 25 frames per second.

Participants were asked to pull hair back, with no make-up or accessories. During the emotion induction procedure, children were informed about the individual variations in emotion expressions and were simply asked to try their best to perform. Participants were constantly reminded to face directly to the front camera.

#### Procedure

Felt experience acting method was used in the current study as the emotion induction method. Expressers performed subsequent emotion conditions following the researcher’s instructions: neutral, interest, disgust, joy, contentment, fear, relief, pride, amusement, surprise, anger, and sadness. The induction procedure was as below. First, the experimenter started the session by telling the child a fictional story of a puppet, in which a specific emotion-eliciting context was embedded in the plot (e.g., disgust, “The puppet was having the breakfast. He drank the expired milk by accident. It stunk and tasted awful.”) During the interaction, the experimenter encouraged the child to think of the puppet’s possible emotion expression under that specific circumstance (e.g., “What kind of expression do you think the puppet would make in response to this?”). After the first question, the experimenter prompted the child to recall and act out his or her own feelings under the same circumstance (e.g., “Take a moment and reflect upon an occasion where you also experienced the puppet’s situation. Could you show me how you expressed your emotion in response to the event?”). To obtain the most naturalistic and diverse emotion responses from children, no rehearsal was required prior to the experiment. Please refer to the appendix for full emotion induction scenarios.

#### Preliminary Processing

Raw video files were saved in RAW or MP4 format after the photography session and were processed *via* Adobe Premiere Pro CC 2018. Clips containing emotion expressions were extracted and converted into JPG images with the best quality. The background of all images was replaced with solid white (RGB255,255,255). Images were cropped into the scale of 4:5 so that the top of each expresser’s head was positioned at the upper 1/6 of the image and the neck aligned with the bottom of the picture.

Preliminary image selection was taken by three experimenters from our research team who initially reviewed all the pictures and agreed on the designated classification of the face-emotion stimulus pairs. For each child expresser, his/her most representative image under each emotion condition was selected, and images with extremely aberrant emotion displays were excluded. Occasionally, more than one images for one single emotion condition from the same child expresser were selected when he/she provided quite different expression presentations, such as open/shut mouth or different head orientations. The result was composed of 712 color photographs of 360 front shots and 352 profile shots and 298 male images and 414 female images (see Table 1). Since we processed front and profile images separately, the image number of these two was slightly different. Then, random sampling was applied in each emotion category, producing 7–9 pictures for each emotion type, and finally included 93 front facing images (46 males, 47 females) from the stimulus set to be evaluated by raters (see Table 2).

**TABLE 1:** Number of pictures of the entire set, classified by gender and face orientation.

**Emotion type**	**Male front**	**Male profile**	**Female front**	**Female profile**	**Total**
Sadness	6	7	11	10	34
Joy	10	11	17	16	54
Anger	5	7	12	10	34
Surprise	19	17	22	22	80
Neutral	22	20	29	29	100
Fear	7	7	8	7	29
Disgust	16	16	18	18	68
Amusement	27	23	28	29	107
Pride	13	13	23	24	73
Contentment	9	9	19	18	55
Relief	7	6	11	12	36
Interest	10	11	11	10	42

**TABLE 2:** Number of pictures, agreement, intensity, and representativeness ratings for the evaluation set, by emotion condition.

**Emotion type**	**Total picture N**	**Ratings N**	**Mean percent agreement for original N**	**N with >50% agreement**	**Mean percent agreement for N with >50% agreement**	**Intensity (*SD*) for N with >50% agreement**	**Representativeness (*SD*) for N with >50% agreement**
Sadness	9	387	89.41%	9	89.41%	4.68 (1.798)	4.81 (1.790)
Joy	8	344	88.66%	8	88.66%	3.90 (1.163)	4.18 (1.332)
Anger	9	387	88.63%	9	88.63%	5.27 (1.465)	5.30 (1.535)
Surprise	7	301	79.40%	7	79.40%	5.22 (1.582)	5.15 (1.562)
Neutral	8	344	77.91%	8	77.91%	4.16 (1.858)	4.57 (1.836)
Fear	7	301	46.84%	3	59.69%	5.29 (1.546)	5.19 (1.694)
Disgust	7	301	19.93%	1	55.81%	5.08 (1.998)	4.87 (2.133)
Amusement	8	344	77.33%	7	82.06%	5.75 (1.217)	5.65 (1.245)
Pride	7	301	75.42%	7	75.42%	5.39 (1.470)	5.35 (1.528)
Contentment	8	344	61.63%	7	64.79%	5.19 (1.536)	5.12 (1.481)
Relief	7	301	45.85%	3	54.26%	5.39 (1.448)	5.51 (1.401)
Interest	8	344	47.09%	3	66.67%	4.93 (1.638)	4.73 (1.491)

### Validation Procedure

#### Participants

Forty-three volunteers were recruited from the internet as validation participants. Raters ranged in age from 18 to 60, 23 females and 20 males, 1 with doctoral degree, 8 with master’s degree, and 28 with bachelor’s degree. Prior to the evaluation, the raters signed the consent form and were informed about the purpose of the study.

#### Procedure

**FIGURE 1:**
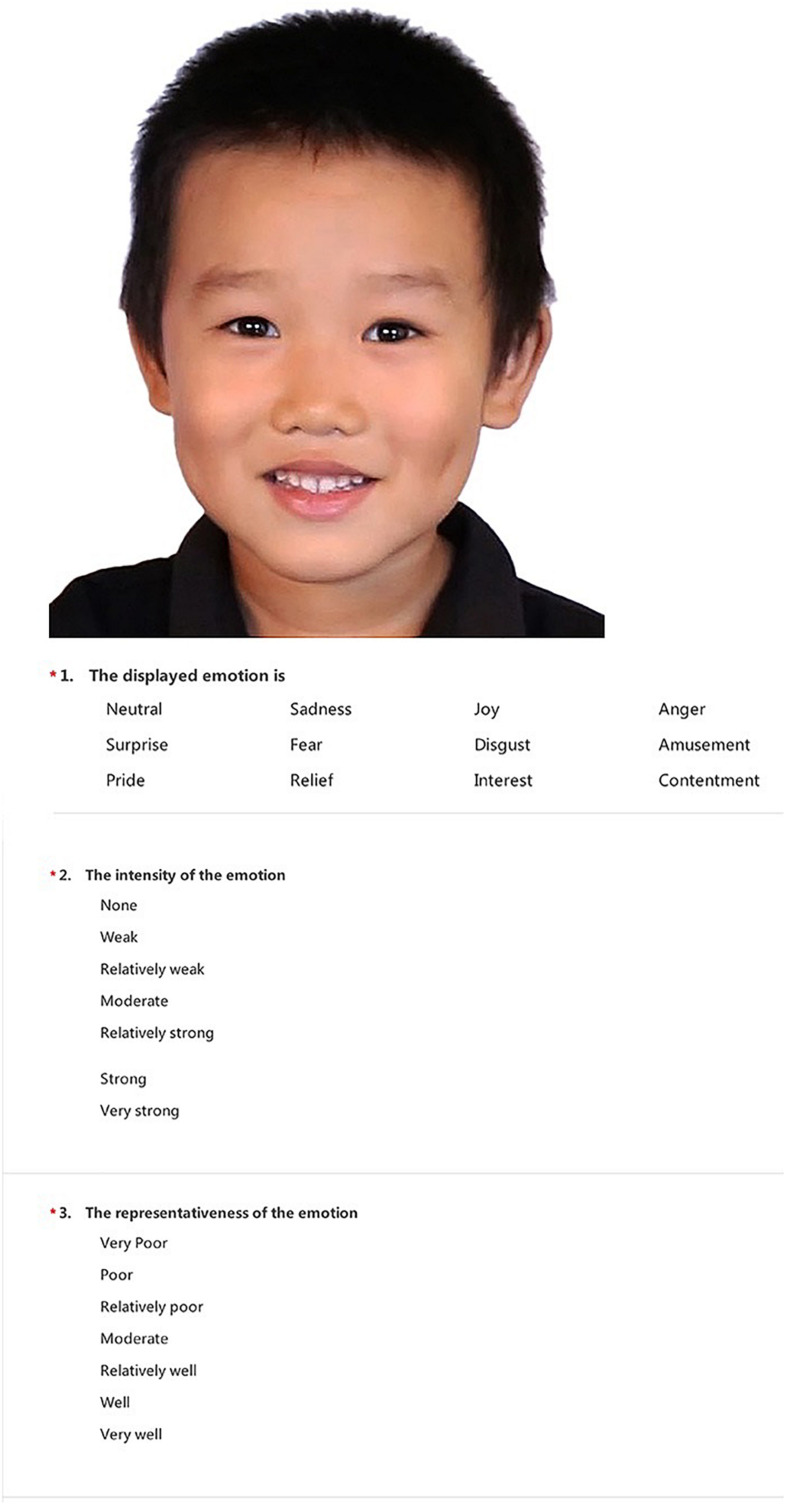
Screenshot of an evaluation page in the questionnaire.

The validation procedure was conducted *via* a secure link to the SoJump questionnaire on a personal laptop or phone. Upon reading a brief introduction describing the rating tasks, the rater entered personal information about gender, age, and education level. A detailed instruction was given before the evaluation started. It included the definition of each emotion condition and the typical scenario when a certain expression occurred, as well as the explanation of each rating tasks with a detailed example. Then, the rater proceeded to evaluate the 93 facial expressions included in the preliminary set. Figure 1 shows an example of the rating tasks for each image. The picture appeared on the top of every evaluation page in the questionnaire and was followed by three multiple choice questions related to the picture presented above. First, to measure the agreement between rating and intended emotion, the rater was asked to choose 1 of the 12 emotions (neutral, sadness, amusement, disgust, anger, surprise, joy, fear, contentment, relief, interest, and pride) that matched the emotion displayed in the picture. Next, the rater was asked to rate the intensity of the emotion on a seven-point scale from weak to very strong. The rater was instructed to choose the intensity level based on the “degree of emotion being expressed” regardless of how confident they felt about the judgment they made on the emotion type in the first question (Egger et al., 2011). Likewise, the rater was then asked to indicate the representativeness of the emotion on a seven-point scale from poorly to very well (Egger et al., 2011). Raters had been instructed to determine how successfully the picture represented the emotion type they had chosen above, despite whether they made the correct choice of intended emotion. The order of images was randomized to avoid the repetitive presentation of the same child expresser or the same emotion type in a row. The entire rating procedure took approximately 40 min. Each rater received a participation fee after the session.

## Results

### Average Agreement Rate

An overall mean agreement rate and agreement rate for each emotion condition were calculated. The overall mean agreement rate was one of the criteria to judge the validity of a stimulus set with other existing ones. It represented the percentage of instances in which a rater considered a picture to be a certain emotion that matched with the intended emotion in the whole stimulus set. The overall mean agreement rate of the original evaluation set was 67.7%.

The average agreement rate for each emotion condition was also calculated and was listed in Table 2. Several influence factors of the agreement rate were examined. Firstly, we examined the gender factor of expressers and raters. There was no significant difference of children’s gender on the agreement rate (*t*(91) = −0.173, *p* = 0.863), with girls’ expressions (*M* = 67.24%, *SD* = 26.27%) and boys’ expressions (*M* = 68.15%, *SD* = 24.10%). There was no significant difference of female raters’ result and male raters’ result (*t*(92) = −0.130, *p* = 0.897), with female raters’ result (*M* = 66.96%, *SD* = 25.78%) and male raters’ result (*M* = 66.78%, *SD* = 25.68%). Secondly, we examined individual emotion type factor. Individual emotion type had a statistically significant effect on the agreement rate (*F*(11,81) = 17.429, *p* < 0.01). *Post hoc* tests showed that the agreement rates of sadness, joy, and anger were statistically better than those of disgust, relief, interest, and fear, respectively (*p*’s < 0.01). In addition, there was no significant interaction between children expresser’s gender and emotion type on the agreement rate (*F*(11,69) = 1.376, *p* = 0.204, η^2^ = 0.180). Lastly, we examined the basic emotion group versus the discrete positive emotion group. There was no statistically significant difference between basic and discrete positive emotions on agreement rate (*t*(83) = 1.778, *p* = 0.79), with basic expressions (*M* = 70.95%, *SD* = 29.20%) and discrete positive expressions (*M* = 61.51%, *SD* = 19.61%).

After the preliminary analysis, a validity criterion was set, and images with less than 50% agreement were excluded. Upon removing 21 pictures (22.5% elimination rate) in total, the acceptable set contains 72 images (45 basic and 27 discrete positive emotions, 37 males and 36 females) for further analysis (see Table 2). After the elimination, the overall mean agreement rate was 78.4%, the average agreement rate of some emotion types had increased, and each emotion type’s agreement rate was over 54% (see Table 2). Firstly, there was also no significant difference of children’s gender on the agreement rate (*t*(70) = 0.621, *p* = 0.537), with girls’ expressions (*M* = 79.53%, *SD* = 15.37%) and boys’ expressions (*M* = 77.31%, *SD* = 15.02%). There was also no significant difference of female raters’ result and male raters’ result (*t*(71) = 0.312, *p* = 0.756), with female raters’ result (*M* = 77.73%, *SD* = 16.02%) and male raters’ result (*M* = 77.27%, *SD* = 16.63%). Secondly, individual emotion type had a statistically significant effect on the agreement rate (*F*(11,60) = 5.373, *p* < 0.01). *Post hoc* tests showed that the agreement rates of sadness and anger were statistically better than that of contentment (*p*’s < 0.01), and that sadness, amusement, anger, surprise, and joy were statistically better than relief (*p*’s < 0.01). Last but not the least, as we predicted, there was a statistically significant difference between basic and discrete positive emotions on agreement rate (*t*(62) = 3.628, *p* < 0.01) in acceptable set, in which basic emotions (*M* = 83.85%, *SD* = 13.62%) were recognized better than discrete positive ones (*M* = 71.06%, *SD* = 14.34%).

### Intensity and Representativeness Ratings

Intensity and representativeness ratings were calculated for those images in acceptable set that were accurately identified by raters based on a seven-point scale and are represented in Table 2. We measured internal consistency (reliability) by calculating Cronbach’s alpha scores between intensity ratings of expression conditions, with overall alpha high (α = 0.929). Intensity and representativeness ratings of all images were highly correlated (Spearman *r* = 0.916, *p* < 0.01). Intensity and representativeness ratings were positively correlated in each emotion condition. There was no significant correlation between agreement rate and intensity ratings (Spearman *r* = −0.128, *p* = 0.285), nor between agreement rate and representativeness ratings (Spearman *r* = 0.005, *p* = 0.969).

Then, we examined the gender factor of expressers and raters. The effect of child expresser’s gender was not significant for intensity ratings (*t*(70) = −1.759, *p* = 0.083), as well as representativeness ratings (*t*(70) = −1.537, *p* = 0.129). The intensity scores of female raters and male raters were statistically different (*t*(71) = −2.504, *p* < 0.05), with female raters’ result (*M* = 4.702, *SD* = 0.763) lower than male raters’ result (*M* = 4.766, *SD* = 0.662). There was no significant difference of female raters’ result and male raters’ result on representativeness ratings (*t*(71) = −0.878, *p* = 0.383).

Thirdly, we examined individual emotion type factor. Emotion type had a significant effect on intensity ratings (*F*(11,60) = 12.412, *p* < 0.01) and also on representativeness ratings (*F*(11,60) = 7.278, *p* < 0.01). The intensity and representativeness scores had a similar pattern that joy and neutral were lower than other emotion types including amusement, anger, surprise, contentment, and pride (*p*’s < 0.01). There was a significant interaction between child expressers’ gender and individual emotion type on the intensity ratings (*F*(10,49) = 2.086, *p* < 0.05, η^2^ = 0.299). The intensity of female expresser’s pride expression was statistically lower than that of male expresser’s pride expression. Among female expressers, most emotion type had higher intensity scores than joy. Among male expressers, amusement, anger, surprise, contentment, and pride all had higher intensity than joy and neutral. Furthermore, there was also a significant interaction between child expressers’ gender and individual emotion type on the representativeness ratings (*F*(10,49) = 2.304, *p* < 0.05, η^2^ = 0.320). The representativeness ratings showed a similar pattern with intensity ratings.

Finally, the intensity ratings for basic emotions and discrete positive emotions were significantly different (*t*(61.390) = −3.711, *p* < 0.01), with higher intensity ratings for discrete positive emotions (*M* = 5.348, *SD* = 0.460) than for basic ones (*M* = 4.807, *SD* = 0.703). In addition, the representativeness ratings for basic emotions and discrete positive emotions were significantly different (*t*(62) = −2.859, *p* < 0.01), with higher representativeness ratings for discrete positive emotions (*M* = 5.281, *SD* = 0.485) than for basic ones (*M* = 4.879, *SD* = 0.601).

### Validity of Individual Images

**FIGURE 2:**
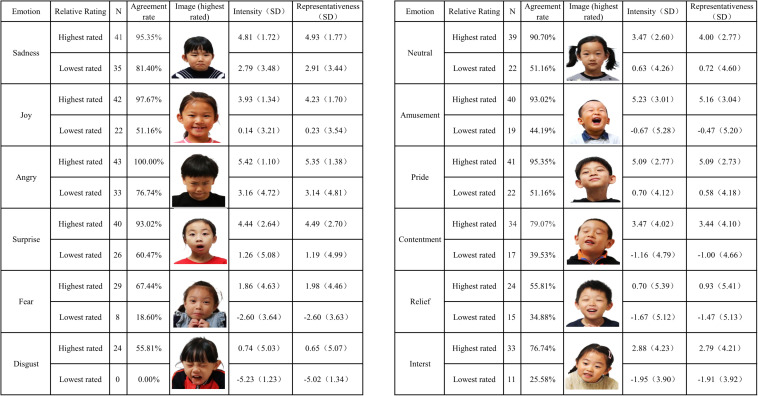
Intensity and representativeness ratings for images with the highest and the lowest percent agreement, by emotion condition; *SD* = standard deviation.

Figure 2 presents the images with the highest and the lowest agreement rate under each emotion condition. Apparently, some emotions were more easily recognized than others. For instance, even the lowest rated image for sadness received 81% agreement, whereas the highest rated image for disgust and relief received only 24% agreement each. Figure 2 also presents the intensity and representativeness scores for these images. According to Egger et al. (2011), if the rater’s label matches with the *a priori* emotion designation, the intensity score accurately reflects the degree of intensity for that particular stimulus and no modification is required. Nevertheless, if the rater mislabels the image, the intensity score should be “penalized” because the stimulus fails to portray the intended emotion. In the latter case, the intensity score is multiplied by −1. Given that a seven-point rating scale was adopted in our study, the intensity and representativeness scores for individual images ranged from −7 to 7. As shown in the figure, the highest rated image for anger received 100% agreement along with the highest intensity and representativeness scores with little standard deviation, which made it the perfect illustrative display for anger. On the opposite, the least accurately identified image for disgust received 0% agreement; therefore, that particular image for disgust received negative scores for both intensity and representativeness ratings. Overall, when an image had an agreement over 90% (i.e., amusement, surprise, sadness, pride, and anger), its corresponding intensity and representativeness scores were also higher (4–5) with smaller standard deviation. On the contrary, images with an agreement lower than 50% failed to represent the intended emotion and received negative intensity and representativeness scores.

### Agreement by Emotion Condition

An overall kappa for images with over 50% agreement rate to estimate the concordance between the raters’ labels and the designated *a priori* emotion was calculated. Among the 72 images in the acceptable set (3,096 observations), we obtained a kappa of *k* = 0.760, *p* < 0.01.

**FIGURE 3:**
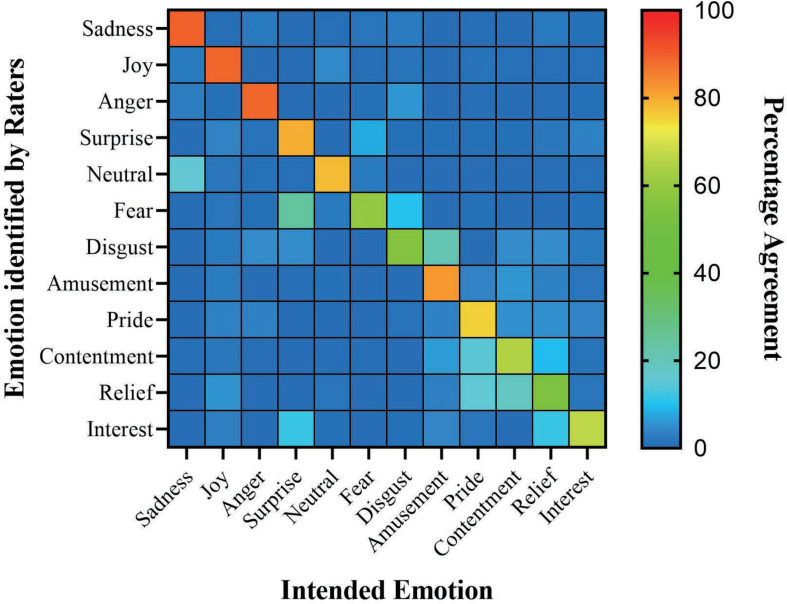
Confusion matrix. Rows represent the emotion identified by raters, and columns represent the designated emotion. The main diagonal shows the degree of agreement between the designated emotion and the raters’ labels, with warmer color representing greater agreement. Off-diagonal entries indicate the confusion across emotions, namely, raters’ misattribution of the intended emotion as a different emotion.

Following the methodology of Dalrymple et al. (2013), we also calculated a confusion matrix (Table 3) to illustrate the degree of agreeability and confusability across emotions. As shown in Figure 3, the main diagonal showed the degree of agreement between the intended emotion and the raters’ labels, with greater agreement represented by warmer color. The off-diagonal entries indicated the raters’ misattribution of the intended emotion with a different emotion, with brighter color indicated more misidentified emotion images.

**TABLE 3:** Confusion matrix of raters’ labels and the designated *a priori* emotion.

		**Emotion identified by raters**											
		**Sadness**	**Joy**	**Anger**	**Surprise**	**Neutral**	**Fear**	**Disgust**	**Amusement**	**Pride**	**Contentment**	**Relief**	**Interest**
**Intended emotion**	Sadness	89.41%	0.26%	2.33%	0.00%	0.52%	1.55%	2.58%	0.00%	0.26%	0.00%	2.33%	0.78%
	Joy	2.33%	88.66%	0.00%	0.00%	1.16%	0.29%	1.45%	3.20%	1.16%	0.58%	0.58%	0.58%
	Anger	2.84%	0.78%	88.63%	0.00%	0.00%	0.78%	5.68%	0.00%	0.26%	0.00%	0.26%	0.78%
	Surprise	0.00%	3.65%	1.00%	79.40%	0.00%	7.97%	0.66%	0.66%	0.66%	0.66%	1.99%	3.32%
	Neutral	15.70%	1.74%	0.87%	0.58%	77.91%	2.33%	0.00%	0.00%	0.00%	0.00%	0.29%	0.58%
	Fear	0.00%	1.55%	0.78%	24.03%	2.33%	59.69%	10.08%	0.00%	0.78%	0.00%	0.00%	0.78%
	Disgust	0.00%	2.33%	4.65%	4.65%	0.00%	0.00%	55.81%	20.93%	0.00%	4.65%	4.65%	2.33%
	Amusement	0.00%	2.66%	0.00%	0.33%	0.66%	0.00%	0.00%	82.06%	3.65%	5.65%	3.32%	1.66%
	Pride	0.00%	3.32%	3.32%	0.00%	0.00%	0.00%	1.00%	3.32%	75.42%	4.98%	4.98%	3.65%
	Contentment	0.33%	1.99%	0.33%	0.00%	0.33%	0.33%	0.00%	6.31%	14.95%	64.78%	9.30%	1.33%
	Relief	0.00%	5.43%	0.00%	0.00%	1.55%	0.00%	0.00%	3.10%	15.50%	18.60%	54.26%	1.55%
	Interest	0.00%	3.10%	0.00%	11.63%	0.78%	0.00%	0.78%	3.88%	1.55%	0.00%	11.63%	66.67%

Considering the agreement rate and misattribution situation, we made a classification similar to the CAFE set (LoBue and Thrasher, 2014) and divided the emotion types into two subsets. Subset A includes pictures of eight emotion types that had relatively high agreement rate and low misattribution, whereas Subset B includes pictures of four emotion types with greater variations (see Table 4). Researchers with interests in easy recognition and little variations would refer to Subset A, and researchers with interests in variations on expressions and ambiguity on recognition would refer to Subset B.

**TABLE 4:** Emotion conditions in subsets.

**Subset A**	**Subset B**
Sadness	Fear
Joy	Disgust
Anger	Relief
Surprise	Interest
Neutral	
Amusement	
Pride	
Contentment	

*Script of emotion induction using the felt experience acting method. 1.Neutral: Please show me a poker face. 2.Interest: The puppet likes candies very much. What kind of expression do you think the puppet would make when he sees so many candies and want them all? Take a moment and reflect upon an occasion where you also experienced the puppet’s situation. Could you show me how you expressed your emotion in response to the event? 3.Disgust: The puppet was having breakfast. He drank the expired milk by accident. It stunk and tasted awful. What kind of expression do you think the puppet would make in response to this? Take a moment and reflect upon an occasion where you also experienced the puppet’s situation. Could you show me how you expressed your emotion in response to the event? 4.Joy: After spilled the milk, mom gave him a candy and the puppet was happy with a smile. What kind of expression do you think the puppet would make in response to this? Take a moment and reflect upon an occasion where you also experienced the puppet’s situation. Could you show me how you expressed your emotion in response to the event? 5. Contentment: The puppet liked his breakfast and ate them all, he was full and satisfied now. What kind of expression do you think the puppet would make in response to this? Take a moment and reflect upon an occasion where you also experienced the puppet’s situation. Could you show me how you expressed your emotion in response to the event? 6. Fear: The puppet was going to school. He saw a fierce dog barking at him and rushed to him, it was dangerous and he was so scared. What kind of expression do you think the puppet would make in response to this? Take a moment and reflect upon an occasion where you also experienced the puppet’s situation. Could you show me how you expressed your emotion in response to the event? 7. Relief: Just as the fierce dog was about to rush in front of him, the owner came over and grabbed the dog to pull it away. The puppet was safe now. What kind of expression do you think the puppet would make in response to this? Take a moment and reflect upon an occasion where you also experienced the puppet’s situation. Could you show me how you expressed your emotion in response to the event? 8. Pride: The puppet arrived at school and was praised by the teacher of good behaviors and became squad leader. What kind of expression do you think the puppet would make in response to this? Take a moment and reflect upon an occasion where you also experienced the puppet’s situation. Could you show me how you expressed your emotion in response to the event? 9. Amusement: Puppet’s friend was telling him a funny joke and made him laugh. What kind of expression do you think the puppet would make in response to this? Take a moment and reflect upon an occasion where you also experienced the puppet’s situation. Could you show me how you expressed your emotion in response to the event? 10. Surprise: Puppet went home and found a lot of friends in his room with a big box of present. It was unanticipated he felt unexpected. What kind of expression do you think the puppet would make in response to this? Take a moment and reflect upon an occasion where you also experienced the puppet’s situation. Could you show me how you expressed your emotion in response to the event? 11. Anger: At the party the puppet was angry and had a fight with someone. What kind of expression do you think the puppet would make in response to this? Take a moment and reflect upon an occasion where you also experienced the puppet’s situation. Could you show me how you expressed your emotion in response to the event? 12. Sadness: The puppet lost the new gift at the party and he was sad. What kind of expression do you think the puppet would make in response to this? Take a moment and reflect upon an occasion where you also experienced the puppet’s situation. Could you show me how you expressed your emotion in response to the event?*

## Discussion

In the current study, we acquired and evaluated the QPFE set, a new facial stimulus set that contains Chinese children’s basic emotions and discrete positive emotions. We collected 712 images featuring 54 5- to 7-year-old Chinese preschoolers with two head orientations: a frontal view and a 45° profile view. To validate the database, 43 untrained adults were recruited online to rate 93 front facing images randomly selected from the full picture set. Two subsets of emotion types were created based on evaluation results.

In our study, the overall mean agreement rate for acceptable set was 78.4%, with a kappa of 0.760, which is comparable with other facial expression stimulus sets. Specifically, the mean agreement is 81% for the NimStim with a kappa of 0.79 (Tottenham et al., 2009), 88% for the POFA (Ekman and Friesen, 1976), 74% for the Japanese and Caucasian Facial Expressions of Emotion (JACFEE) (Matsumoto and Ekman, 1989), 89% for the Karolinska Directed Emotional Faces (KDEF) (Flykt et al., 1998), and overall 76.4% mean agreement for five stimulus sets including NimStim and POFA (Palermo and Coltheart, 2004). The mean agreement for stimulus sets including children are 66% for the CAFE full set, 81% for Subset A (LoBue and Thrasher, 2014), 86% for the Dartmouth Database of Children’s Faces (with a kappa of 0.78) (Dalrymple et al., 2013), 86% for the DEFSS (Meuwissen et al., 2017), 90.4% for the NIMH-ChEFS (with a kappa of 0.86), and 94.8% for the acceptable set of the NIMH-ChEFS (with a kappa of 0.94) (Egger et al., 2011). There are probably two reasons why the QPFE seems to have a slightly lower agreement rate. Firstly, as we predicted, our validation results showed that basic emotions were identified better than discrete positive emotions. The inclusion of more nuanced discrete positive emotions posed challenges for the raters to capture the subtle differences among emotions. Secondly, considering the felt experience acting method adopted in the current study, unlike the prototype posing method used in other picture sets for children emotion induction, we expected greater expression variations in children’s emotion displays and, thus, is disadvantageous for the emotion labeling task (Bänziger et al., 2011).

Agreement ratings from previous stimulus sets showed that sadness, anger, and fear were almost unanimously regarded as the least accurately identified emotions (Ekman and Friesen, 1976; Biehl et al., 1997; Wang and Markham, 1999; Calvo and Lundqvist, 2008; Tottenham et al., 2009; Gong et al., 2011). In the current study, sadness and anger had the highest average agreement, respectively. This incongruent finding was possibly due to the inclusion of discrete positive emotions. That is, given that sadness and anger lack of the idiosyncratic “Duchenne smile” of most positive emotions (except for awe), thus the two emotions could be easily distinguished from others in the stimulus set. In fact, the basic emotion group that contains fewer positive emotions did receive an overall better agreement rate than discrete positive emotions in our study. Among discrete positive emotions in our study, amusement had the highest average agreement rate, along with relatively high intensity and representativeness scores. Bänziger et al. (2011) also found that amusement was the best recognized among 12 emotions.

In terms of intensity and representativeness ratings, our results resembled previous findings in showing a high correlation between the two parameters of each emotion condition (Langner et al., 2010; Meuwissen et al., 2017). For instance, amusement, pride, and anger all received high intensity and representativeness ratings. This indicated that emotions with inherently higher intensity might also be more indicative or representative of the emotion type. This was a similar discovery with Prada et al. (2018) that clarity was strongly and positively associated with genuineness and with intensity. In addition, as suggested by Livingstone and Russo (2018), pictures with strong intensity were rated as more genuine than normal intensity productions. Further, consistent with the NIMH-ChEFS and the DEFSS (Egger et al., 2011; Meuwissen et al., 2017), sadness, despite receiving a low-intensity score, was the most accurately identified emotion in the current study. Similar with joy, receiving the lowest intensity score, it was also the most accurately identified. It resembled what Hess et al. (1997) discovered that relatively low-intensity happy faces had high recognition accuracy. This indicated that the level of emotional intensity might not be associated with the accurate identification of emotions. Indeed, the current study revealed a lack of significant correlation between intensity and agreement ratings or between representativeness and agreement ratings, in contrast with previous studies (Palermo and Coltheart, 2004).

We examined the gender factor of both child expressers and raters. Expressers’ gender had significant influence on neither agreement ratings nor intensity and representativeness ratings, meaning that male and female preschoolers’ expressions were not significantly different. This was consistent with some research studies (LoBue and Thrasher, 2014; Coffman et al., 2015). Raters’ gender had no significant influence on agreement ratings and representativeness ratings. However, we found a significant difference of intensity ratings on raters’ gender, meaning that female raters intended to rate lower intense than male raters. It was quite opposite to Dalrymple et al.’s (2013) findings, who found that female raters rated more intense than male raters. The difference was due to the age range of expressers or ethnic matters merit further exploration.

Emotion type, however, had a significant influence on both agreement ratings and intensity and representativeness ratings, which was consistent with most expression stimulus sets (LoBue and Thrasher, 2014; Coffman et al., 2015; Meuwissen et al., 2017). In our research, basic emotions were recognized better than discrete positive ones, whereas the intensity and representativeness ratings of discrete positive emotions were better than those of basic ones. This might indicate that raters may be confused about positive emotion types due to the subtle differentiation in between; however, they might find that a positive emotion image expressed comparatively high intensity and could represent the intended mood.

The misattribution in the current study would also provide some new perspective for future research. For the first one, same as discovered by Langner et al. (2010), surprise, fear, and disgust contained an overlap misattribution with each other. Disgust has been shown in previous studies to be easily confused with other negative emotions, such as anger or sadness (Bänziger et al., 2011), as well as contempt (Langner et al., 2010). Consistent with the CFAPS, disgust received the lowest agreement in the current study and was most frequently mislabeled as amusement. There were possibly three reasons to explain the situation. Firstly, it could be probably due to the similar expressive displays shared between the two emotions. Disgust contains nose wrinkle, upper lip raise, and lips apart (van der Schalk et al., 2011), whereas amusement contains intense smiles and open jaws (Sauter, 2017). In the QPFE, we observed that most preschoolers’ disgust expressions contained outstretched tongues, which could also be a symbol of hilarity. Secondly, according to Grossard et al. (2018), during the expression induction session, children might feel embarrassed when they had to produce negative emotions, so that they tended to laugh. This was exactly what happened during our session. This might influence the final expression of disgust with some clue of laugh. Finally, it was also possible that facial expression was not the most efficient cue for discriminating positive and negative emotions with relatively high intensities. For instance, Aviezer et al. (2012) showed that during peak intensities of emotions, isolated bodies, but not faces, provided better cues for people in identifying the valence of certain emotions. For the second one, relief, contentment, and pride were often confused in the current study. Specifically, relief was commonly mislabeled as contentment and pride, pride was mislabeled as relief, and contentment was mislabeled as pride and relief. This misattribution was possibly due to the similar facial muscle movement and the core relational themes shared by the three emotions. All three expressions contained a smile with slightly closed eyes and slightly open mouth (Krumhuber and Scherer, 2011; Mortillaro et al., 2011; Campos et al., 2013). In addition, according to Campos et al. (2013), all three expressions shared the similar core relational themes that contained the feeling of satisfied, safe, and relaxed. In addition, as suggested by Shiota et al. (2017), postural expressions, but not faces, might provide better cues for the identification of certain positive emotions. For instance, pride usually consists of sitting up straight and holding one’s head up (Tracy and Robins, 2008), amusement usually consists of a unique body shake, a tilt of the head (Campos et al., 2013), and satisfaction usually consists of a motionless body and a small but rapid nod (Campos et al., 2013). In addition, in a recent research (Mortillaro and Dukes, 2018), facial dynamics and body representations were considered to be the critical elements for positive emotion differentiation. These could all be taken into further considerations.

## Limitations

Firstly, the child expressers’ age range in the current study was between 5 and 7 years old. Compared with other facial expression stimulus sets, it was quite a small range. In this study, we were more interested in facial expression induction for preschoolers, which was younger than 7. Furthermore, former research studies showed Chinese children’s ability for understanding emotions stabilized at the age of 5 (Wang et al., 2010). However, it would indeed reduce the diversity of children’s facial expressions, and the data we have now might not represent the expression display situation of other age groups. A larger range of age would be considered for future work.

Secondly, adults only were recruited as evaluaters in the current study. With the purpose of validating the new obtained emotional expression set, adults would be more understandable to facial emotion recognition and validation procedures. Recruiting children as raters to evaluate their same-aged peer’s facial expressions, comparing the efficiency with adult raters and thereby further examining the effect of age on facial emotion recognition, will be our next move.

## Conclusion

In this paper, we presented the QPFE set, the first independent Chinese children’s facial stimulus set. The database includes five discrete positive emotions (interest, contentment, relief, pride, and amusement) in addition to the traditionally well-studied basic emotions in two subsets. The overall mean agreement rate and kappa were highly comparable with existing facial stimulus sets. The felt experience acting method successfully induced some positive emotions among the preschoolers, with the finding that certain positive emotions (i.e., amusement and pride) received better identification than others. In general, the data would contribute to the local and cross-cultural emotion-face research, especially enriching the absence of literature on positive emotions and children’s emotion expressions.

## Data Availability Statement

The raw data supporting the conclusions of this article will be made available by the authors, without undue reservation.

## Ethics Statement

The studies involving human participants were reviewed and approved by Institute of Psychology, Chinese Academy of Sciences. Written informed consent to participate in this study was provided by the participants’ legal guardian/next of kin. Written informed consent was obtained from the individual(s), and minor(s)’ legal guardian/next of kin, for the publication of any potentially identifiable images or data included in this article.

## Author Contributions

GZ and YZ designed the experiments and wrote the manuscript. JC carried out the experiments, analyzed the experimental data, and wrote the manuscript. All authors contributed to the article and approved the submitted version.

## Conflict of Interest

The authors declare that the research was conducted in the absence of any commercial or financial relationships that could be construed as a potential conflict of interest.

## References

[B1] Adolphs R. (2002). Recognizing emotion from facial expressions: psychological and neurological mechanisms. *Behav. Cogn. Neurosci. Rev.* 1 21–62. 10.1177/1534582302001001003 17715585

[B2] Ambadar Z. Cohn J. F. Reed L. I. (2009). All smiles are not created equal: morphology and timing of smiles perceived as amused, polite, and embarrassed/nervous. *J. Nonverbal Behav.* 33 17–34. 10.1007/s10919-008-0059-5 19554208 PMC2701206

[B3] Aviezer H. Trope Y. Todorov A. (2012). Body cues, not facial expressions, discriminate between intense positive and negative emotions. *Science* 338 1225–1229. 10.1126/science.1224313 23197536

[B4] Bänziger T. Mortillaro M. Scherer K. (2011). Introducing the Geneva multimodal expression corpus for experimental research on emotion perception. *Emotion* 12 1161–1179. 10.1037/a0025827 22081890

[B5] Benoit K. E. McNally R. J. Rapee R. M. Gamble A. L. Wiseman A. L. (2012). Processing of emotional faces in children and adolescents with anxiety disorders. *Behav. Change* 24 183–194. 10.1375/bech.24.4.183

[B6] Biehl M. Matsumoto D. Ekman P. Hearn V. Heider K. Kudoh T. (1997). Matsumoto and Ekman’s Japanese and Caucasian facial expressions of emotion (JACFEE): reliability data and cross-national differences. *J. Nonverbal Behav.* 21 3–21. 10.1023/A:1024902500935

[B7] Blakemore S. J. (2008). The social brain in adolescence. *Nat. Rev. Neurosci.* 9 267–277. 10.1038/nrn2353 18354399

[B8] Calvo M. G. Lundqvist D. (2008). Facial expressions of emotion (KDEF): identification under different display-duration conditions. *Behav. Res. Methods* 40 109–115. 10.3758/brm.40.1.109 18411533

[B9] Campos B. Wang S. W. Plaksina T. Repetti R. L. Schoebi D. Ochs E. (2013). Positive and negative emotion in the daily life of dual-earner couples with children. *J. Fam. Psychol.* 27 76–85. 10.1037/a0031413 23421835

[B10] Camras L. Perlman S. Fries A. Pollak S. (2006). Post-institutionalized Chinese and Eastern European children: heterogeneity in the development of emotion understanding. *Int. J. Behav. Dev.* 30 193–199. 10.1177/0165025406063608

[B11] Coffman M. C. Trubanova A. Richey J. A. White S. W. Kim-Spoon J. Ollendick T. H. (2015). Validation of the nimh-chefs adolescent face stimulus set in an adolescent, parent, and health professional sample. *Int. J. Methods Psychiatr. Res.* 24 275–286. 10.1002/mpr.1490 26359940 PMC5103077

[B12] Dalrymple K. A. Gomez J. Duchaine B. (2013). The dartmouth database of children’s faces: acquisition and validation of a new face stimulus set. *PLoS One* 8:e79131. 10.1371/journal.pone.0079131 24244434 PMC3828408

[B13] Egger H. L. Pine D. S. Nelson E. Leibenluft E. Ernst M. Towbin K. E. (2011). The NIMH child emotional faces picture set (NIMH-ChEFS): a new set of children’s facial emotion stimuli. *Int. J. Methods Psychiatr. Res.* 20 145–156. 10.1002/mpr.343 22547297 PMC3342041

[B14] Ekman P. (1992). Facial expressions of emotion: new findings, new questions. *Psychol. Sci.* 3 34–38. 10.1111/j.1467-9280.1992.tb00253.x

[B15] Ekman P. Friesen W. (1978). *Facial Action Coding System (FACS): A Technique for the Measurement of Facial Action.* Palo Alto, CA: Consulting Psychologists Press.

[B16] Ekman P. Friesen W. V. Tomkins S. S. (1971). Facial affect scoring technique: a first validity study. *Semiotica* 3 37–58. 10.1515/semi.1971.3.1.37

[B17] Ekman P. Friesen W. V. (1976). *Pictures of Facial Affect.* Palo Alto, CA: Consulting Psychological Press.

[B18] Flykt A. Lundqvist D. Öhman A. (1998). The Karolinska directed emotional faces (KDEF). *CD ROM from Department of Clinical Neuroscience, Psychology Section, Karolinska Institutet* 91–630. 10.1017/S0048577299971664 10194972

[B19] Fredrickson B. L. (1998). What good are positive emotions? *Rev. Gen. Psychol.* 2 300–319. 10.1037/1089-2680.2.3.300 21850154 PMC3156001

[B20] Gong X. Huang Y. X. Wang Y. Luo Y. J. (2011). Revision of the Chinese facial affective picture system. *Chin. Ment. Health J.* 25 40–46. 10.3969/j.issn.1000-6729.2011.01.011

[B21] Griskevicius V. Shiota M. N. Neufeld S. L. (2010). Influence of different positive emotions on persuasion processing: a functional evolutionary approach. *Emotion* 10 190–206. 10.1037/a0018421 20364895

[B22] Grossard C. Chaby L. Hun S. Pellerin H. Bourgeois J. Dapogny A. (2018). Children facial expression production: influence of age, gender, emotion subtype, elicitation condition and culture. *Front. Psychol.* 9:446. 10.3389/fpsyg.2018.00446 29670561 PMC5894457

[B23] Hess U. Blairy S. Kleck R. E. (1997). The intensity of emotional facial expressions and decoding accuracy. *J. Nonverbal Behav.* 21 241–257. 10.1023/a:1024952730333

[B24] Hoehl S. Brauer J. Brasse G. Striano T. Friederici A. D. (2010). Children’s processing of emotions expressed by peers and adults: an fMRI study. *Soc. Neurosci.* 5 543–559. 10.1080/17470911003708206 20486013

[B25] Krumhuber E. G. Scherer K. R. (2011). Affect bursts: dynamic patterns of facial expression. *Emotion* 11 825–841. 10.1037/a0023856 21707163

[B26] Langner O. Dotsch R. Bijlstra G. Wigboldus D. H. J. Hawk S. T. van Knippenberg A. (2010). Presentation and validation of the Radboud Faces Database. *Cogn. Emot.* 24 1377–1388. 10.1080/02699930903485076

[B27] Leppänen J. Moulson M. Vogel-Farley V. Nelson C. (2007). An ERP study of emotional face processing in the adult and infant brain. *Child Dev.* 78 232–245. 10.1111/j.1467-8624.2007.00994.x 17328702 PMC2976653

[B28] Livingstone S. R. Russo F. A. (2018). The Ryerson audio-visual database of emotional speech and song (ravdess): a dynamic, multimodal set of facial and vocal expressions in North American English. *PLoS One* 13:e0196391. 10.1371/journal.pone.0196391 29768426 PMC5955500

[B29] LoBue V. Thrasher C. (2014). The child affective facial expression (CAFE) set: validity and reliability from untrained adults. *Front. Psychol.* 5:1532. 10.3389/fpsyg.2014.01532 25610415 PMC4285011

[B30] Louie J. Oh B. Lau A. (2013). Cultural differences in the links between parental control and children’s emotional expressivity. *Cultur. Divers. Ethnic Minor. Psychol.* 19 424–434. 10.1037/a0032820 23834255

[B31] Macchi Cassia V. (2011). Age biases in face processing: the effects of experience across development. *Br. J. Psychol.* 102 816–829. 10.1111/j.2044-8295.2011.02046.x 21988386

[B32] Macchi Cassia V. Pisacane A. Gava L. (2012). No own-age bias in 3-year-old children: more evidence for the role of early experience in building face-processing biases. *J. Exp. Child Psychol.* 113 372–382. 10.1016/j.jecp.2012.06.014 22857798

[B33] Marusak H. A. Carre J. M. Thomason M. E. (2013). The stimuli drive the response: an fMRI study of youth processing adult or child emotional face stimuli. *Neuroimage* 83 679–689. 10.1016/j.neuroimage.2013.07.002 23851324

[B34] Matsumoto D. (1992). American-Japanese cultural differences in the recognition of universal facial expressions. *J. Cross Cult. Psychol.* 23 72–84. 10.1177/0022022192231005

[B35] Matsumoto D. Ekman P. (1989). American–Japanese cultural differences in judgments of facial expressions of emotion. *Motiv. Emot.* 13 143–157. 10.1007/BF00992959

[B36] Meuwissen A. S. Anderson J. E. Zelazo P. D. (2017). The creation and validation of the developmental emotional faces stimulus set. *Behav. Res. Methods* 49 960–966. 10.3758/s13428-016-0756-7 27325165 PMC5173446

[B37] Miki K. Honda Y. Takeshima Y. Watanabe S. Kakigi R. (2015). Differential age-related changes in N170 responses to upright faces, inverted faces, and eyes in Japanese children. *Front. Hum. Neurosci.* 9:263. 10.3389/fnhum.2015.00263 26082700 PMC4451338

[B38] Mortillaro M. Dukes D. (2018). Jumping for joy: the importance of the body and of dynamics in the expression and recognition of positive emotions. *Front. Psychol.* 9:763. 10.3389/fpsyg.2018.00763 29867704 PMC5962906

[B39] Mortillaro M. Mehu M. Scherer K. R. (2011). Subtly different positive emotions can be distinguished by their facial expressions. *Soc. Psychol. Pers. Sci.* 2 262–271. 10.1177/1948550610389080

[B40] Nelson E. E. McClure E. B. Monk C. S. Zarahn E. Leibenluft E. Pine D. S. (2003). Developmental differences in neuronal engagement during implicit encoding of emotional faces: an event-related fMRI study. *J. Child Psychol. Psychiatry* 44 1015–1024. 10.1111/1469-7610.00186 14531584

[B41] Palermo R. Coltheart M. (2004). Photographs of facial expression: accuracy, response times, and ratings of intensity. *Behav. Res. Methods Instrum. Comput.* 36 634–638. 10.3758/bf03206544 15641409

[B42] Prada M. Garrido M. V. Camilo C. Rodrigues D. L. (2018). Subjective ratings and emotional recognition of children’s facial expressions from the CAFE set. *PLoS One* 13:e0209644. 10.1371/journal.pone.0209644 30589868 PMC6307702

[B43] Sabatinelli D. Fortune E. E. Li Q. Siddiqui A. Krafft C. Oliver W. T. (2011). Emotional perception: meta-analyses of face and natural scene processing. *Neuroimage* 54 2524–2533. 10.1016/j.neuroimage.2010.10.011 20951215

[B44] Sauter D. A. (2017). The nonverbal communication of positive emotions: an emotion family approach. *Emot. Rev.* 9 222–234. 10.1177/1754073916667236 28804510 PMC5542129

[B45] Sauter D. A. Eisner F. Ekman P. Scott S. K. (2010). Cross-cultural recognition of basic emotions through nonverbal emotional vocalizations. *Proc. Natl. Acad. Sci. U.S.A.* 107 2408–2412. 20133790 10.1073/pnas.0908239106 PMC2823868

[B46] Scherer K. R. Bänziger T. (2010). “On the use of actor portrayals in research on emotional expression,” in *A Blueprint for Affective Computing: A Sourcebook and Manual*, eds Scherer K. R. Bänziger T. Roesch E. (Oxford: Oxford University Press).

[B47] Scherer K. R. Heiner E. (2007). Are facial expressions of emotion produced by categorical affect programs or dynamically driven by appraisal? *Emotion* 7 113–130. 10.1037/1528-3542.7.1.113 17352568

[B48] Shiota M. N. Campos B. Oveis C. Hertenstein M. J. Simon-Thomas E. Keltner D. (2017). Beyond happiness: building a science of discrete positive emotions. *Am. Psychol.* 72 617–643. 10.1037/a0040456 29016167

[B49] Shiota M. N. Neufeld S. L. Yeung W. H. Moser S. E. Perea E. F. (2011). Feeling good: autonomic nervous system responding in five positive emotions. *Emotion* 11 1368–1378. 10.1037/a0024278 22142210

[B50] Taylor M. J. Batty M. Itier R. J. (2004). The faces of development: a review of early face processing over childhood. *J. Cogn. Neurosci.* 16 1426–1442. 10.1162/0898929042304732 15509388

[B51] Todd R. Lewis M. Meusel L. A. Zelazo P. (2008). The time course of social-emotional processing in early childhood: ERP responses to facial affect and personal familiarity in a Go-Nogo task. *Neuropsychologia* 46 595–613. 10.1016/j.neuropsychologia.2007.10.011 18061633

[B52] Tottenham N. Tanaka J. W. Leon A. C. Mccarry T. Nurse M. Hare T. A. (2009). The NimStim set of facial expressions: judgments from untrained research participants. *Psychiatry Res.* 168 242–249. 10.1016/j.psychres.2008.05.006 19564050 PMC3474329

[B53] Tracy J. L. Robins R. W. (2008). The nonverbal expression of pride: evidence for cross-cultural recognition. *J. Pers. Soc. Psychol.* 94 516–530. 10.1037/0022-3514.94.3.516 18284295

[B54] Tracy J. L. Robins R. W. Schriber R. A. (2009). Development of a FACS-verified set of basic and self-conscious emotion expressions. *Emotion* 9 554–559. 10.1037/a0015766 19653779

[B55] Tsai J. L. Knutson B. Fung H. H. (2006). Cultural variation in affect valuation. *J. Pers. Soc. Psychol.* 90 288–307. 10.1037/0022-3514.90.2.288 16536652

[B56] van der Schalk J. Hawk S. T. Fischer A. H. Doosje B. (2011). Moving faces, looking places: validation of the Amsterdam dynamic facial expression set (ADFES). *Emotion* 11 907–920. 10.1037/a0023853 21859206

[B57] Wang L. Markham R. (1999). The development of a series of photographs of Chinese facial expressions of emotion. *J. Cross Cult. Psychol.* 30 397–410. 10.1177/0022022199030004001

[B58] Wang Z. H. Tian B. Shi C. D. Cui X. R. (2010). The developmental characteristics of recognition and free labeling of facial expression of emotions in 3 6-year-old children. *Chin. Psychol. Sci.* 33 325–328.

